# Attachment anxiety and smartphone addiction among university students during confinement: Teacher–student relationships, student–student relationships and school connectedness as mediators

**DOI:** 10.3389/fpubh.2022.947392

**Published:** 2022-08-04

**Authors:** Wen Zhang, Fangzhou Zhou, Qingyu Zhang, Zhixuan Lyu

**Affiliations:** ^1^School of Journalism and Culture Communication, Zhongnan University of Economics and Law, Wuhan, China; ^2^Institute of Communication Studies, Communication University of China, Beijing, China; ^3^School of Accounting, Zhongnan University of Economics and Law, Wuhan, China; ^4^School of Journalism and Communication, Xiamen University, Xiamen, China

**Keywords:** COVID-19, attachment anxiety, smartphone addiction, student-student relationships, teacher-student relationships, school connectedness

## Abstract

Learning at home during the COVID-19 confinement might affect students' relationships with their peers, teachers, and schools and increase the possibility of smartphone addiction. We hypothesized that attachment anxiety directly and indirectly affects smartphone addiction, with teacher–student relationships, student–student relationships, and school connectedness as mediators. The participants were 999 university students from different regions of China. The results showed that six of the paths were significant except the one between student–student relationships and smartphone addiction. Also, the association between attachment anxiety and smartphone addiction was mediated by teacher–student relationships and school connectedness not but student–student relationships. The current study highlights the mediating effect of school connectedness and teacher–student relationships in the multiple mediation model, and suggests that universities can alleviate the risk of smartphone addiction in distance teaching by cultivating good teacher–student relationships and strengthening students' sense of belonging to their schools.

## Introduction

On December 31, 2019, Chinese health officials initially notified the World Health Organization's (WHO) Beijing office that a cluster of pneumonia cases of unclear origin had appeared in Wuhan, the capital of Central China's Hubei Province (1). On February 11, 2020, WHO formally designated the virus COVID-19, short for “coronavirus disease 2019” (2). COVID-19 caused a large-scale outbreak of new pneumonia, which shut down Wuhan for 76 days. Since early 2020, COVID-19 has emerged and spread rapidly on a global scale, with serious impacts on public health and social life in various countries (3). Until 5 July 2022, it has killed 6 million people and infected 500 million more around the world (4). Many universities around the world have stopped traditional in-person teaching and have implemented online teaching models (5). As the pandemic continues to spread, the landscape of education is experiencing rapid changes with an undefined time course (6), and learning at home may remain normalized for some time to come. People are spending much more time on smartphone than they did pre-outbreak, with nearly 30% online for more than 5 h a day (7). But it is unclear whether the risk of smartphone addiction might increase when students were learning at home during confinement.

Smartphone addiction is a phenomenon where individuals indulged in smartphone-based activities and had strong desire and dependency on their smartphones (8). Several studies have introduced other terms such as problematic smartphone use (9), mobile phone addiction (10), addictive smartphone behavior (11). In the current study, we decided to use the term “smartphone addiction” to describe the condition.

As a common source of behavioral addiction, smartphone addiction can cause severe damage to both physical and mental health. University students nowadays are digital natives who have grown up surrounded by smartphones (12). However, the key changes in their psychological and brain development have not yet been completed, making them more vulnerable to the impact of smartphone addiction (9). Extensive use of smartphones may lead to sleep disorders (13) and may also affect emotions and cognition. A high level of smartphone addiction is associated with anxiety, depression, impulsivity, and other types of mental distress among university students (14).

Therefore, numerous studies have focused on the causes of smartphone addiction. Depression, low self-esteem, loneliness, and shyness may contribute to smartphone addiction (15, 16). As a personality trait, attachment style is also an important predictor of smartphone addiction. An anxious attachment style has been shown to be positively related to smartphone addiction (17). Many studies have reported the indirect effects of attachment style on smartphone addiction, with typical mediators including emotions (18, 19), psychological distress (20–22), and relationships (10, 23).

Different from western university students, Chinese university students are accommodated uniformly by their schools, with four to eight students sharing the same dormitory and often hanging out together. In addition, students often participate in school activities as a unit of class, with a strong collective. As adolescents grow into independent adults, physical proximity to parental attachment figures becomes less important (24). Thus, compared with primary and middle school students, the role of university students' relationships at school exceeds that of family relationship. The quarantine policies and home-based learning brought by COVID-19 have led to social distancing and damage to human relationships (25), maybe with greater influence on ones with anxious attachment style. The level of attachment anxiety in Chinese university students has been on the rise in recent years (26), and individuals with high attachment anxiety tend to have more conflicts in their relationships (27). Primary human relationships include interpersonal relationships and organizational relationships (28). The need to quarantine at home during the pandemic has led to students spending long periods far away from the university campus, during which time they mainly communicate with their teachers and schoolmates online, which might influence their relationships with peers, teachers, and schools. Then, the damage of relationships might drive them to depend alternatively on smartphones for possible consolation (10). However, no study has explored how these “relationships” mediate the association between attachment anxiety and smartphone addiction during confinement. We focused on Flores' (29) theory, which states that the higher the level of attachment anxiety, the more difficult it is to satisfy the relationships with others, leading to various types of addiction. We anticipated that attachment anxiety would influence smartphone addiction via relationship factors such as interpersonal and organizational relationships. Therefore, in current study, we explored how teacher–student relationships, student–student relationships, and school connectedness mediate the relationship between attachment anxiety and smartphone addiction among university students. We sought to build a conceptual framework based on the attachment theory: Chinese university students accustomed to collective life with attachment anxiety tend to have poor relationships with their peers, teachers and schools during the confinement period, and therefore lead to smartphone addiction. Based on the results, we aimed to develop a better understanding of the possible causes of university students' smartphone addiction during the confinement, to provide tangible solutions to alleviate the problem, and also to establish a foundation from which we and other researchers could make further investigation of the impact of attachment anxiety on smartphone addiction.

### Anxious attachment style and smartphone addiction

Addiction is a manifestation of attachment disorder, and there is an inverse relationship between addiction and healthy interpersonal attachment (29). Attachment styles have frequently been referred to in studies of addictive behavior, and a growing number of studies (30) have examined the influence of attachment styles on technology addiction (20).

Attachment theory was proposed by the psychologist John Bowlby in the 1960s (31). The theory conceptualizes the tendency of humans to establish strong emotional ties with intimate people. According to the theory, individuals who experience unwilling separation and loss are at risk of various forms of emotional distress and personality disorder, including anxiety, anger, depression, and emotional detachment (32). Attachment styles can be divided into secure attachment and insecure attachment, and the latter can be divided into anxious attachment and avoidant attachment (33). People with an anxious attachment style have a hyperactive attachment system and tend to seek constant support and comfort. People with an avoidant attachment style, in contrast, have a deactivated attachment system that leads to the inhibition of psychological and social relationship needs, which means that they deliberately maintain a clear distance from others (34). Unlike attachment avoidance, people with an anxious attachment style are more impacted during the confinement, because they are more invested in the relationship out of fear of being abandoned and alone (33).

Attachment anxiety has been reported to cause intensive and dysfunctional Internet and social media use, and is closely related to smartphone addiction (17, 35). People with attachment anxiety are afraid of being abandoned and are likely to engage in intimacy-seeking, relationship-related disclosure behaviors (36). If their relationships with family members and peers (30) do not satisfy their relationship needs, anxiety about being abandoned or rejected by others may drive them to use smartphones for comfort (10), which eventually increases the risk of addiction (19). Therefore, we theorized that students who are unable to return to campus or meet their teachers and peers at school during home quarantine find it hard to satisfy their attachment needs, which increases the probability of smartphone addiction. More specifically, we hypothesized that attachment anxiety directly affects smartphone addiction during the confinement, leading to our first hypothesis.

**H1:** During the confinement period, attachment anxiety is positively related to smartphone addiction among university students.

### Teacher–student relationships and student-student relationships as mediators of smartphone addiction

Numerous studies have examined how interpersonal relationships may contribute to smartphone addiction (37, 38). Individuals with high attachment anxiety are reported to get less social support and be less satisfied with the interpersonal relationships they build. They frequently worry that their friends will abandon them, so they try to be as emotionally close to others as possible. They could turn to smartphones for safety as a result of unhappy relationships (39). For example, individuals with high attachment anxiety frequently use their cell phones to contact others in order to maintain stable relationships (40). They are more likely to be on social media and to be concerned about how others perceive them (41). Student-student and teacher–student relationships are important types of interpersonal relationships among university students (42). When emerging adults leave home for university, there is a gradual shift from parental attachment to peer attachment (43), which means that students are more likely to turn to their peers and teachers to satisfy their attachment needs (27). Although teachers' work is mainly focused on education, providing care and emotional support is also an important part of their role (44). Therefore, although a teacher–student relationship is not a complete attachment bond, it can create a certain sense of security for the student (45). In terms of student–student relationships, an insecure attachment style is negatively related to perceived peer support (46). Attachment anxiety can predict the quality of university students' peer relationships, which is manifested through friendship quality and the level of loneliness (27).

Meanwhile, the quality of interpersonal relationships is closely related to smartphone addiction. Studies have demonstrated both direct (47) and indirect (48) effects of teacher–student relationships on smartphone addiction. Specifically, perceived support from teachers can reduce problematic smartphone use by promoting students' self-esteem (47). Deviant peer affiliation, representing poor student–student relationships, is positively related to Internet game addiction (49). For the same reason, healthy student–student relationships are negatively associated with smartphone addiction (50). Based on our review of the relevant literature, we hypothesized that attachment anxiety would negatively predict a good interpersonal relationship and a tendency toward smartphone addiction:

**H2a:** During the confinement period, attachment anxiety is negatively related to good teacher–student relationships, which in turn is negatively related to smartphone addiction. In other words, teacher-student relationships mediate the association between attachment anxiety and smartphone addiction.
**H2b:** During the confinement period, attachment anxiety is negatively related to good student–student relationships, which in turn is negatively related to smartphone addiction. In other words, student-student relationships mediate the association between attachment anxiety and smartphone addiction.

### School connectedness as a mediator of smartphone addiction

School connectedness is a composite variable describing students' psychological sense of safety and membership to their school environment (51). The importance of school connectedness has been suggested because students need meaningful and supportive relationships outside of their family, and such bonds usually occur in the school environment (52). Attachment anxiety directly affects students' connectedness and life satisfaction, and individuals with insecure attachment styles are likely to experience difficulties in fulfilling their need to belong (53). Students with positive school connectedness enjoy their school life and have better sense of belonging in school (54). School connectedness has been shown to be a salient predictor of students' psychological wellbeing and prosocial behavior (55).

Also, students' feelings about school act as prominent predictors of smartphone addiction. For example, problematic Internet use is lower among students who show appreciation for their school than among those who do not (56); lower school connectedness is related to increased Internet game addiction (57); and maladjustment in school is associated with smartphone addiction (58). Given that school connectedness is likely to be weaker when students are far away from campus, we propose the following hypotheses in the context of the confinement. We hypothesized that school connectedness acts as a mediator in the relationship between anxiety attachment and smartphone addiction. This is based on the significant relationship between anxiety attachment and school connectedness, school connectedness and smartphone addiction, and anxiety attachment and smartphone addiction.

**H2c:** During the confinement period, attachment anxiety is negatively related to school connectedness, which in turn is negatively related to smartphone addiction. In other words, student-student relationships mediate the association between attachment anxiety and smartphone addiction.

**Figure 1:**
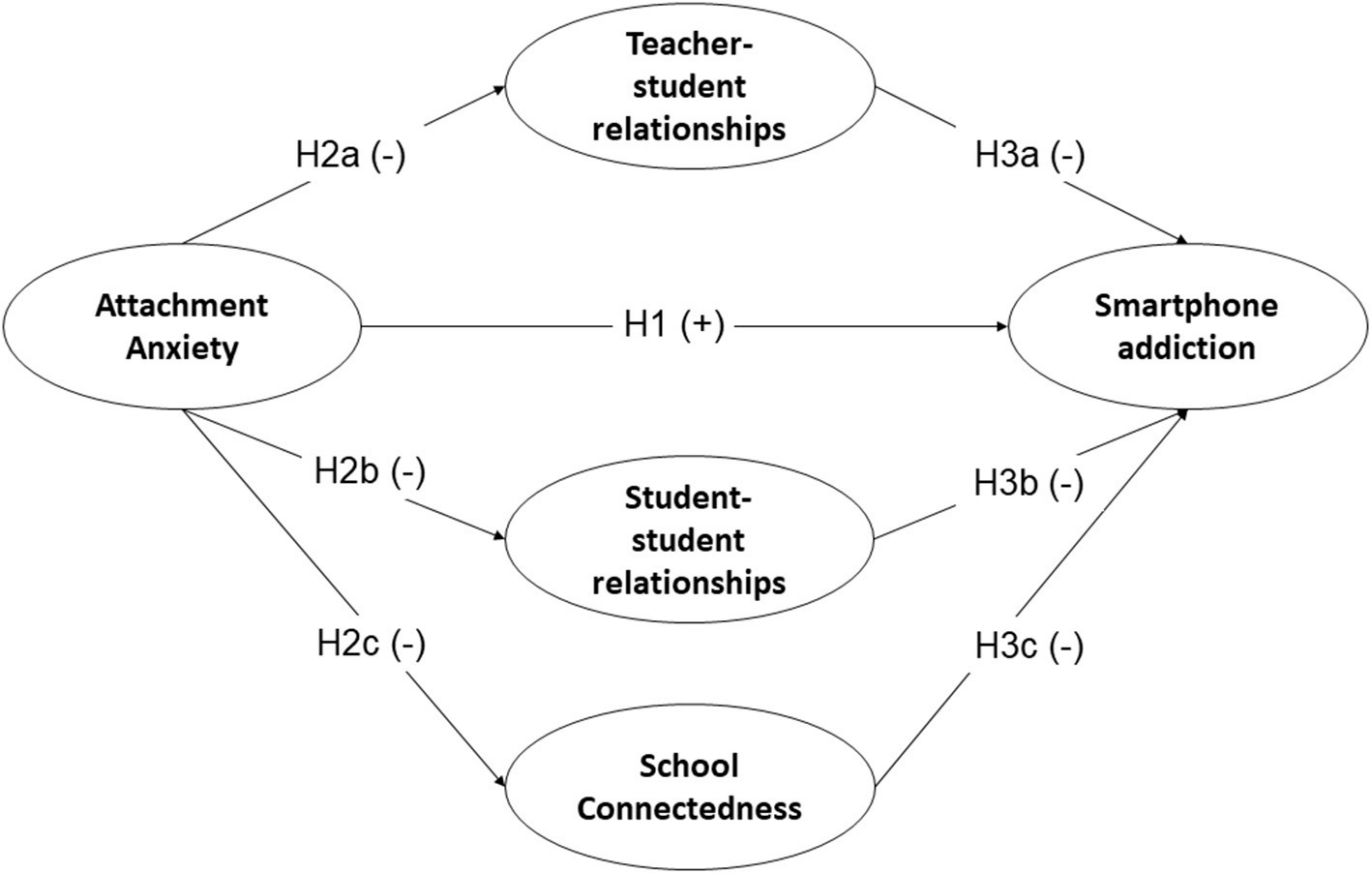
Hypothetical model. (+) Means a hypothesized positive association, and (-) means a hypothesized negative association.

The hypothetical model is shown in Figure 1.

## Materials and methods

### Participants

In the worst year of the epidemic in China, most universities took quarantine measures. Due to the confinement policy, a two-stage sampling survey method was used, combining probability and non-probability sampling. Based on geographic position and socioeconomic development, China is divided into four major regions: Northeastern region, Eastern region, Central region, and Western region. Then, five typical administrative cities were selected from each region. The participants were recruited online *via* messages sent to undergraduate students through WeChat in 2020. To ensure the validity of the research, screening was performed to ensure the participants (1) were Chinese university students, (2) were studying at a university that conducted confinement policy requiring home study, and (3) possessed a smartphone. After screening, 1,052 eligible participants remained, but data from 53 of them were excluded because their response times were too short or their responses to different items were too similar, according to the standard practice for questionnaire-based research. Eventually, 999 participants (384 male and 615 female, M*age* = 20.05, SD*age* = 1.175, age range 17–29) provided valid data. We obtained informed consent forms from all the participants and one from a 17-year-old participant's parents. This study was approved by the Research Ethics Committee of the corresponding's author's university.

The participants completed a questionnaire designed to collect information on demographic variables, attachment anxiety, teacher–student relationships, student–student relationships, school connectedness, and smartphone addiction. After completing the questionnaire, the participants received modest monetary compensation in return for their contribution to the study. 52.7% of the participants were junior and 26.2% were sophomore. More than half of the participants studied in Northeastern region and Eastern region. 74.6% of the participants experienced home learning exceeding 4 months. The detailed demographic information is shown in Table 1.

**Table 1:** Demographic information of the participants (*N* = 999).

**Demographics**		**Frequency**	**Percentage**
Gender	Male	384	38.4
	Female	615	61.6
Grade	Freshman	99	9.9
	Sophomore	262	26.2
	Junior	526	52.7
	Senior	112	11.2
School location	Northeastern region	302	30.2
	Eastern region	326	32.6
	Central region	161	16.1
	Western region	210	21.0
Home learning duration	<2 months	96	9.6
	2–3 months	158	15.8
	4 months or more	745	74.6

### Measures

#### Covariates

The information about participants' gender, age, online course duration, and whether to use smartphone to access online courses were collected as covariates.

#### Attachment anxiety

Based on previous research on attachment styles (10), attachment anxiety was measured by four items selected from the Adult Attachment Scale (AAS). AAS was developed by Collins and Read (59) based on a scale created by Hazan and Schaeffer and updated in 1996 (e.g., “I often worry about being abandoned by my friends.”) In China, this scale has been proven to be reliable (60). The participants indicated their agreement with each item on a 7-point Likert scale from 1 (not at all true) to 7 (always true). A higher score means more severe attachment anxiety. In the current study, Cronbach's alpha for this scale was 0.925, indicating good reliability.

#### Teacher–student relationships during the confinement

Teacher–student relationships were measured by three items adapted from Pianta's (61) Student–Teacher Relationship Scale (STRS) and the Inventory of Parent and Peer Attachment (IPPA) developed by Armsden and Greenberg (62), which include items such as “During the confinement period, I could tell my problems and troubles to at least one teacher at school.” Initially designed to evaluate peer and parent relationships, the IPPA has also been adapted to assess students' relationships with their teachers (22). The participants indicated their agreement with each item on a 7-point Likert scale from 1 (not at all true) to 7 (always true). A higher score means better teacher–student relationships. In the current study, Cronbach's alpha for this scale was 0.844.

#### Student–student relationships during the confinement

Student–student relationships were measured by three items adapted from the Inventory of Parent and Peer Attachment (62) (e.g., “Although we couldn't meet during the confinement period, my friends at school could tell when I was upset about something.”). This scale was revised in 2011 and has been proven to be reliable in China (63). Responses were given on a 7-point Likert scale from 1 (not at all true) to 7 (always true) based on the participants' cognition of student–student relationships during the confinement. A higher score means better student–student relationships. In the current study, Cronbach's alpha for this scale was 0.885.

#### School connectedness during the confinement

School connectedness was measured using three items adapted from Resnick et al's. (64) School Connectedness Scale and the Program for International Student Assessment (PISA) student survey (Chinese version) (65). The items measure students' engagement at school and whether or not they feel part of their schools (e.g., “The lack of school atmosphere during the confinement period made me feel awkward and out of place.”). The PISA questionnaire was used and demonstrated to be reliable in a previous study of students' connectedness to school (66). Responses were given on a 7-point Likert scale from 1 (not at all true) to 7 (always true) based on the participants' sense of belonging to school during the confinement. All three items were reverse coded, and a higher score means greater school connectedness. In the current study, Cronbach's alpha for this scale was 0.682.

#### Smartphone addiction during the confinement

Smartphone addiction was measured using five items adapted from Leung's (67) Mobile Phone Addiction Index (MPAI) and Young's (68) screening questionnaire (e.g., “During the confinement period, I used my smartphone to make myself feel better when I was feeling down.”). Items were answered on a 7-point Likert scale from 1 (not at all true) to 7 (always true). A higher score means more severe smartphone addiction. In the current study, Cronbach's alpha was 0.890.

### Data analysis

We conducted descriptive statistical analysis using SPSS version 19 to examine the characteristics of the participants and the measured constructs. Structural equation modeling (SEM) was performed with Mplus version 7 to test the relationships among the latent constructs.

Following a two-stage procedure (69), we first performed confirmatory factor analysis (CFA) to evaluate the measurement model, which verified the associations between the latent constructs and the observable variables by assessing the reliability and validity of the measurement tools.

Second, the structural equation model tested the relationships among the constructs by examining the standardized path coefficients. We performed a bootstrap procedure to test the mediation effects of our hypothetical model, as this has been shown to be a valid and powerful method of verifying mediation effects (70).

We also evaluated the fitness of the model using the chi-square test, degree of freedom (df), standardized root mean square residual (SRMR), Tucker–Lewis index (TLI), comparative fit index (CFI), and root mean square error of approximation (RMSEA). TLI and CFI values > 0.90 and RMSEA values < 0.08 indicate an acceptable model fit (71).

## Results

### Examination of the measurement model

To evaluate the measurement model, we used Cronbach's alpha and composite reliability (CR) values to examine the reliability of the constructs. According to Fornell and Larcker (72), CR values above 0.700 represent good composite reliability. In the current study, Cronbach's alpha ranged from 0.682 to 0.925, and the CR values ranged from 0.784 to 0.927, suggesting that the reliability of the latent constructs was good or acceptable (see Table 2).

**Table 2:** Descriptive statistics, reliability, and validity of constructs.

	**Mean**	**SD**	**Cronbach's α**	**CR**	**AVE**
Attachment anxiety	2.736	1.543	0.925	0.927	0.762
Teacher–student relationships	3.891	1.536	0.844	0.880	0.710
Student–student relationships	4.511	1.484	0.885	0.904	0.759
School connectedness	4.435	1.476	0.682	0.784	0.548
Smartphone addiction	3.608	1.478	0.890	0.896	0.637

CR, composite reliability; and AVE, average variance extracted.

To test the validity of the measurement model, convergent and discriminant validity were evaluated. Convergent validity was evaluated by the average variance extracted (AVE) of each construct. The AVE values ranged from 0.548 to 0.762 (see Table 2), and all were above the recommended value of 0.500 (72), thus indicating good convergent validity. To evaluate discriminant validity, we compared the square root of the AVE with the absolute values of the correlation coefficients between each construct and the other constructs. According to Table 3, the square roots of the AVE of each construct were larger than the absolute values of the correlation coefficients between constructs. Therefore, discriminant validity was also acceptable.

**Table 3:** Construct correlations and discriminant validity.

	**1**	**2**	**3**	**4**	**5**
1. Attachment anxiety	**0.873**				
2. Teacher–student relationships	−0.077*	**0.842**			
3. Student–student relationships	−0.212**	0.435**	**0.871**		
4. School connectedness	−0.312**	0.254**	0.191**	**0.740**	
5. Smartphone addiction	0.428**	−0.188**	−0.135**	−0.432**	**0.798**

^**^p < 0.01, ^*^p < 0.05, and the square roots of the AVE were shown in bold.

### Examination of the structural model

**Figure 2:**
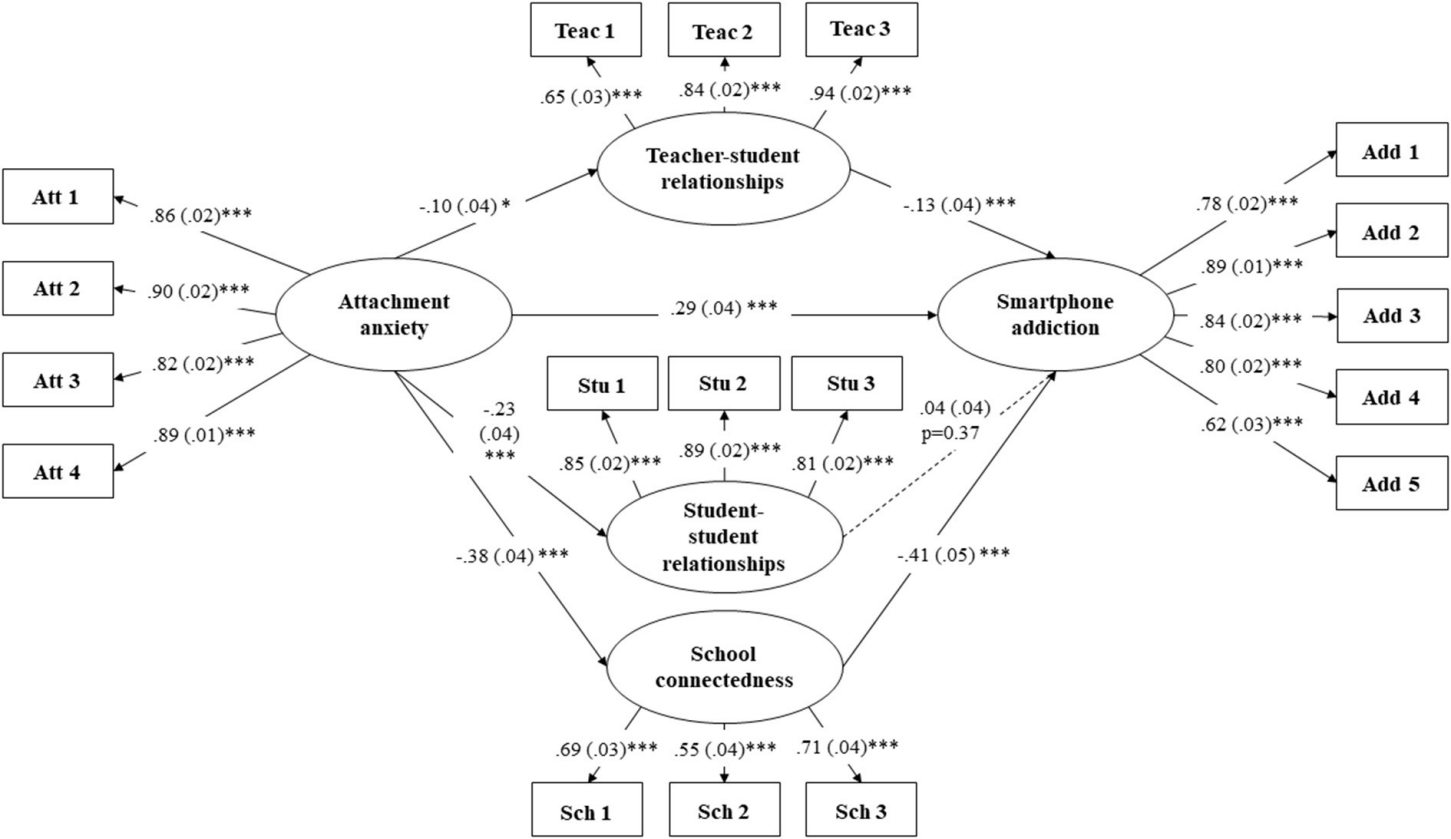
Multiple mediation model. Path values are the standardized path coefficients (standard errors); ***p < 0.001, *p < 0.05. Gender, age, online course duration, and whether to use smartphone to access online courses were controlled.

The structural model was analyzed to test the relationships between the constructs. The hypothetical model and path coefficients (standard errors) are shown in Figure 2. According to the path coefficients, attachment anxiety was positively related to smartphone addiction (β = 0.29, *p* < 0.001), thus confirming **H1**. The paths between attachment anxiety and teacher–student relationships (**H2a**, β = −0.10, *p* = 0.014), attachment anxiety and student–student relationships (**H2b**, β = −0.23, *p* < 0.001), and attachment anxiety and school connectedness (**H2c**, β = −0.38, *p* < 0.001) were all statistically significant, thus supporting **H2a**, **H2b**, and **H2c**. Teacher–student relationships (**H3a**, β = −0.13, *p* = 0.001) and school connectedness (**H3c**, β = −0.41, *p* < 0.001) were both negatively associated with smartphone addiction, thus confirming **H3a** and **H3c**. However, the path between student–student relationships and smartphone addiction (**H3b**, β = 0.04, *p* = 0.370) was not significant, thus **H3b** was not confirmed. Only one of the seven paths in the model was not significant. The hypothetical model provided a good fit to the data (χ^2^ = 1011.215; df = 196; SRMR = 0.081; TLI = 0.914; CFI = 0.925; RMSEA = 0.065).

### Verification of the mediation effects

The bootstrap method was used to test the indirect effects of attachment anxiety on smartphone addiction. The indirect effects of the three mediators (teacher–student relationships, student–student relationships, and school connectedness) in the hypothetical model were estimated with 10,000 randomly assigned samples from the original data (Table 4). The mediation effects of teacher–student relationships and school connectedness were both significant because the 95% confidence intervals did not include zero, thus confirming **H4a** and **H4c**. However, the mediation effect of student–student relationships was not significant as the 95% confidence interval included zero (*p* = 0.383); thus, **H4b** was not confirmed. Comparison of the mediation effects among the three constructs showed that the contrast between the mediation effects of teacher–student relationships and school connectedness was significant (*p* < 0.001), implying the existence of different mediation effects on the relationship between attachment anxiety and smartphone addiction. Table 5 shows that most of our hypotheses were verified.

**Table 4:** Specific indirect effects.

	**Point estimate**	**Product of coefficients**			**Bootstrap (10,000 samples)**			
					**BC 95% CI**		**Percentile 95% CI**	
		**S.E**.	**Est./S.E**.	**P**	**Lower**	**Upper**	**Lower**	**Upper**
**Indirect effects**								
Teac	0.012	0.006	1.880	*	0.003	0.028	0.002	0.026
Stu	−0.008	0.009	−0.872	0.383	−0.027	0.009	−0.027	0.009
Sch	0.144	0.026	5.556	***	0.100	0.202	0.098	0.199
Total	0.148	0.028	5.381	***	0.100	0.208	0.098	0.206
**Contrasts**								
Teac vs. Stu	−0.020	0.012	−1.605	0.108	−0.048	0.001	−0.046	0.002
Teac vs. Sch	−0.133	0.026	−5.022	***	−0.190	−0.087	−0.188	−0.085
Stu vs. Sch	−0.152	0.028	−5.446	***	−0.213	−0.104	−0.210	−0.101

Teac, teacher–student relationships; Stu, student–student relationships; Sch, school connectedness; BC, bias corrected. ^***^p < 0.001, ^*^p < 0.05.

**Table 5:** Hypotheses and conclusions.

**Hypotheses**	**Conclusion**
**H1:** Attachment anxiety → Smartphone addiction	Confirmed
**H2a:** Attachment anxiety → Teacher–student relationships	Confirmed
**H2b:** Attachment anxiety → Student–student relationships	Confirmed
**H2c:** Attachment anxiety → School connectedness	Confirmed
**H3a:** Teacher–student relationships → Smartphone addiction	Confirmed
**H3b:** Student–student relationships → Smartphone addiction	Not confirmed
**H3c:** School connectedness → Smartphone addiction	Confirmed
**H4a:** Attachment anxiety → Teacher–student relationships → Smartphone addiction	Confirmed
**H4b:** Attachment anxiety → Student–student relationships → Smartphone addiction	Not confirmed
**H4c:** Attachment anxiety → School connectedness → Smartphone addiction	Confirmed

## Discussion

The COVID-19 pandemic may have normalized home quarantine. For Chinese university students who are accustomed to collective life, especially those with high attachment anxiety, the confinement policy would affect their relationships with peers, teachers and schools. Then, smartphone addiction might be used to make up for the lack of these relationships. However, there has been no research on whether learning during the confinement period, which affects both interpersonal and organizational relationships, is associated with the severity of problematic smartphone use. Based on attachment theory and previous studies, the current study established a multiple mediation model between attachment anxiety and smartphone addiction among university students during the COVID-19 pandemic.

Our results showed that attachment anxiety was positively associated with smartphone addiction, demonstrating that students with higher attachment anxiety are more likely to develop smartphone addiction. This finding is in line with that of Han et al. (17), who also found that attachment anxiety was positively related to mobile phone addiction. Attachment anxiety was also associated with smartphone addiction through multiple mediators. We believe that university students' excessive smartphone use serves as a kind of emotional compensation for their failed interpersonal and organizational relationships during home quarantine. According to previous research, emotional compensation occurs when people seek to adjust their emotions to balance what they perceive to be an inappropriate response from others (73). This finding can help to shed light on the present study: when students were unable to gain attachment satisfaction in their relationships with peers, teachers, and schools, they were highly likely to turn to their smartphones for emotional compensation.

### The mediating effect of teacher–student relationships during the COVID-19 confinement

We found that attachment anxiety was negatively associated with good teacher–student relationships. Meanwhile, teacher–student relationships had a negative influence on smartphone addiction, which is in line with the study by Peng et al. (47), which found that teachers' support for students' autonomy reduced students' problematic smartphone use. Teacher–student relationships were a significant mediator in our study. These results suggest that during the COVID-19 pandemic, university students with high attachment anxiety tend to have poor relationships with their teachers, which increases the potential for problematic smartphone use.

The mediation effect of teacher–student relationships implies that unlike face-to-face regular periods, students with a high level of attachment anxiety tend to lack interactions and bidirectional emotional exchanges with their teachers during home quarantine. They try to compensate for their sense of emptiness and insecurity through the use of smartphones, and excessive use can easily lead to smartphone addiction. The mediating effect demonstrates that teacher–student relationships have an important role in preventing smartphone addiction. The findings also imply that if teachers provide care and support to enhance their relationships with students, students will be less likely to develop smartphone addiction during the confinement.

### The mediating effect of student–student relationships during the COVID-19 confinement

As expected, attachment anxiety was negatively associated with student–student relationships. This suggests that in the confinement students with high attachment anxiety are likely to have poor relationships with their school peers. Previous studies (74) have found that student–student relationships are negatively correlated with addictive behavior, whereas healthy student–student relationships can alleviate addiction. However, similar to Ihm's (75) study, we found no significant association between student–student relationships and smartphone addiction; therefore, the mediating effect was also non-significant. This suggests that even though university students with attachment anxiety tend to have problematic student–student relationships, they are unlikely to compensate for the lack of companionship by excessively using their smartphones.

We believe that student–student relationships failed to mediate the relationship between attachment anxiety and smartphone addiction because the policy of home quarantine served as an external attribution source. In the absence of face-to-face communication, students with attachment anxiety have a reasonable explanation for the lack of concern and understanding from their peers, and the sense of loss caused by online alienation from school peers seems more acceptable than feeling isolated offline. In addition, without face-to-face communication, individuals can still perceive the presence of their school peers through multiple channels, such as “liking” and commenting on their social media posts. In contrast, during the confinement, online courses are almost the only way for students to perceive the existence of their teachers and schools, and to maintain their relationships through interaction with them.

Individuals with attachment anxiety are prone to show aggressive behavior after being treated unfairly (76). They also hold contradictory attitudes toward their attachment figures, exhibiting dependent and rejecting behaviors simultaneously (30). Therefore, if the need for security is unsatisfied, individuals with attachment anxiety tend to behave in extreme ways and try to impose their psychological pain on their attachment objects. Students who perceive neglect and threat from their school peers during home quarantine may respond in this way, which is actually facilitated by distance and online communication. However, when individuals believe that they have been unfairly treated by their schools and teachers, it is difficult for them to retaliate and they may turn to their smartphones to vent their emotions. Furthermore, this study only focused on student–student relationships in the school context. During the confinement, students can compensate for the loss of companionship at school by strengthening their relationships with their neighborhood peers. Hence, it is understandable that no significant relationship was found between student–student relationships and smartphone addiction.

### The mediating effect of school connectedness during the COVID-19 confinement

Similar to teacher–student relationships, school connectedness was negatively correlated with attachment anxiety and smartphone addiction. School connectedness also significantly mediated the relationship between attachment anxiety and smartphone addiction. These findings indicate that students with high attachment anxiety tend to lack school connectedness during the confinement, which could lead to smartphone addiction. In contrast, university students with strong school connectedness are less likely to develop smartphone addiction in the confinement period.

When away from school during quarantine, students have little direct contact with their schools. The lack of interaction and participation in school life can lead to university students feeling detached from their schools. Students in Chinese universities are provided with uniform housing, and they typically socialize in dorms of four to eight people. Students who board on campus can focus more on their studies, form better relationships with others, and feel more connected to the school (77). The lack of parental love and care may also be partially made up for by the campus community. The safety and health of students with high attachment anxiety, as well as the development of wholesome living and studying practices, are also ensured by school policies, peer supervision, and teachers' guidance (78). It is critical to cultivate a student-first campus culture and a diversified boarding life, where students can express emotions, learn to collaborate, and deal with peer relationships through numerous school activities (79). Generally speaking, Chinese universities have complete, student-oriented facilities with varied activities and caring living arrangements to provide emotional support for students. However, the quarantine policies and home-based learning brought by COVID-19 forced students to leave school suddenly. If universities are not able to provide good services, varied activities, and more supervision during the confinement via the internet, there is a greater chance that students will become addicted to their smartphones. If schools could introduce more measures to increase the sense of belonging during the confinement period, smartphone addiction could be alleviated to a great extent.

### Contrasting mediation effects

A multiple mediation model was established in this study. Examination of the mediation effects revealed significant differences between teacher–student relationships and school connectedness, and student–student relationships and school connectedness. The contrasting mediation effects indicate that these two mediators relieve smartphone addiction rather differently.

The literature review suggested that teacher–student relationships often appear to be part of the school relationship. Relationships with teachers and school peers have been used to measure students' relational maladjustment in school (58). In the current study, we examined school connectedness in the same dimension as interpersonal relationships with teachers and school peers. The significant difference in mediation effects between school connectedness and teacher–student relationships confirms our expectation that teacher–student relationships can operate as an independent mediator. Teachers and schools alleviate smartphone addiction differently. Positive relationships with teachers can provide students with warmth and security, which may satisfy their psychological needs and in turn relieve their Internet addiction (48). Likewise, encouraging school appreciation (56) and strengthening school connectedness can offer students a sense of participation and security that could help to prevent smartphone addiction.

Although student–student relationships did not have a significant mediation effect, there was a significant difference in mediation effects between student–student relationships and school connectedness. This highlights the importance of the mediating effect of school connectedness in the multiple mediation model, and suggests that a strong sense of school connectedness is more helpful than student–student relationships in alleviating students' mobile phone addiction.

## Conclusion

In the context of the COVID-19 pandemic, we explored the multiple paths between attachment anxiety and smartphone addiction using the framework of attachment theory. A multi-mediation model was established, and we found that attachment anxiety both directly and indirectly affected smartphone addiction among university students. Teacher–student relationships and school connectedness served as significant mediators of smartphone addiction. However, student–student relationships was not associated with smartphone addiction during the confinement period.

The literature on attachment styles and addiction focuses mainly on interpersonal, especially parental and peer, relationships (80). Although school connectedness has also been widely mentioned (54, 81), most studies have merely emphasized the interaction between the sense of connectedness and interpersonal relationships (82, 83). As far as we are aware, few studies have taken school connectedness as an organizational relationship and examined it in the same dimension as interpersonal relationships. Our findings show that attachment anxiety is not only related to interpersonal relationships but can also influence organizational relationships.

Students with high attachment anxiety use smartphones to compensate for feelings of emptiness and loss. If individuals with attachment anxiety find it difficult to perceive personal relationships or school connectedness in reality, they may become addicted to smartphones, specifically activities centered on relationships (e.g., online games or SNS).

Psychologists specializing in the prevention and treatment of smartphone addiction are likely to focus on the obsession with smartphones, unaware that students may be suffering from high attachment anxiety, perceiving poor personal relationships, and lacking school connectedness during their confinement on COVID-19. Excessive smartphone use can be attributed to maladaptive coping strategies or relational factors; therefore, prevention and treatment should concentrate on relational factors such as interventions to improve teacher-student relationships and school connectedness, and adaptive coping strategies. Recognizing and managing those relational factors, in the future, may help to alleviate the problem of smartphone addiction among university students when they are unable to attend school. On the one hand, students should be aware that smartphone addiction is more likely to occur in distance learning or e-learning and consciously avoid the threat. On the other hand, universities should pay attention to the efficiency and practicability of distance teaching, focus on cultivating good relationships between teachers and students, and enhance students' sense of belonging to their schools. Creating a sense of security and participation for students, so that they may fully perceive the existence of their teachers and schools even when they are away from campus, should help to reduce the possibility of smartphone addiction to a great extent.

Despite our innovations and contributions, a few limitations of this study should be taken into account. First, the use of an online self-report survey led to some limitations, although an offline study was not possible because of the pandemic. For instance, the participants were not selected through probability sampling only; rather, the questionnaires were collected online and the results were screened. In addition, self-report bias is always a possibility with self-report questionnaires. Second, this was a cross-sectional study based on a retrospective design and was thus subject to the inherent limitations associated with retrospective analyses. Compared with prospective studies, the cause–effect relationships are uncertain in a retrospective design. The variables cannot be manipulated and observed systematically, which may lead to inaccurate conclusions. Third, the data were collected from a single country and are only representative of Chinese university students; thus the study lacks a multicultural perspective in the context of the global pandemic. Whether the research results can be applied to other cultural contexts remains unknown. Fourth, we did not examine relationships other than teacher–student relationships, student–student relationships, and school connectedness in the present study. For example, parental relationships and the presence of other cohabitants may also have an effect on the conclusion during home study, which could be incorporated in future studies. Also, assessing the severity of smartphone addiction using only five items is another limitation of the present study.

## Data availability statement

The datasets generated and analysed during the current study are not publicly available due to the consideration of participants' privacy, but are available from the corresponding author on reasonable request.

## Ethics statement

The studies involving human participants were reviewed and approved by the Research Ethics Committee of Zhongnan University of Economics and Law. Written informed consent to participate in this study was provided by the participants' legal guardian/next of kin.

## Author contributions

FZ and WZ analyzed data and wrote the main manuscript text. QZ and ZL were responsible for investigation and data curation. All authors reviewed the manuscript. All authors contributed to the article and approved the submitted version.

## Funding

This study was supported by the 2021 project of “the Publicity Department of Hubei Provincial Party Committee and Zhongnan University of Economics and Law Co-construction Journalism School” (Grant number: 2020-2-2-05).

## Conflict of interest

The authors declare that the research was conducted in the absence of any commercial or financial relationships that could be construed as a potential conflict of interest.

## Publisher's note

All claims expressed in this article are solely those of the authors and do not necessarily represent those of their affiliated organizations, or those of the publisher, the editors and the reviewers. Any product that may be evaluated in this article, or claim that may be made by its manufacturer, is not guaranteed or endorsed by the publisher.

## Appendix

**Table A1:** Measurement items and factor loading.

**Construct**	**Item**	**Loading**
Attachment anxiety	Att 1. I often worry about being abandoned by my friends.	0.872
	Att 2. I often worry that my friends don't really care about me.	0.898
	Att 3. I find others are reluctant to get as close as I would like.	0.824
	Att 4. I often worry that my friends will not want to stay with me.	0.895
Teacher–student relationships during the confinement	Teac 1. During the confinement period, I could tell my problems and troubles to at least one teacher at school.	0.797
	Teac 2. During the confinement period, most teachers made me feel that they were concerned about my studies.	0.849
	Teac 3. During the confinement period, most teachers made me feel that they were concerned about my daily life.	0.879
Student–student relationships during the confinement	Peer 1. Although I couldn't meet my schoolmates during the confinement period, they still cared about how I was feeling.	0.863
	Peer 2. During the confinement period, I could count on my friends at school when I needed to get something off my chest.	0.903
	Peer 3. Although we couldn't meet during the confinement period, my friends at school could tell when I was upset about something.	0.847
School connectedness during the confinement	Sch 1. During the confinement period, I didn't feel like I belonged at school. (Reverse scored)	0.776
	Sch 2. I wish I had studied at another school during the confinement period. (Reverse scored).	0.697
	Sch 3. The lack of school atmosphere during the confinement period made me feel awkward and out of place. (Reverse scored)	0.746
Smartphone addiction during the confinement	Add 1. During the confinement period, I used my smartphone for increased amounts of time to achieve satisfaction.	0.821
	Add 2. During the confinement period, I used my smartphone to make myself feel better when I was feeling down.	0.863
	Add 3. During the confinement period, I attempted to spend less time on my smartphone but was unable to.	0.861
	Add 4. During the confinement period, I felt anxious and lost when I hadn't turned on my smartphone for some time.	0.804
	Add 5. During the confinement period, my family and friends complained about my use of the smartphone.	0.614
